# A20 Restricts Wnt Signaling in Intestinal Epithelial Cells and Suppresses Colon Carcinogenesis

**DOI:** 10.1371/journal.pone.0062223

**Published:** 2013-05-06

**Authors:** Ling Shao, Shigeru Oshima, Bao Duong, Rommel Advincula, Julio Barrera, Barbara A. Malynn, Averil Ma

**Affiliations:** 1 Department of Medicine, University of California San Francisco, San Francisco, California, United States of America; 2 Department of Advanced Therapeutics in Gastrointestinal Disease, Tokyo Medical and Dental University, Tokyo, Japan; University of Aberdeen, United Kingdom

## Abstract

Colon carcinogenesis consists of a multistep process during which a series of genetic and epigenetic adaptations occur that lead to malignant transformation. Here, we have studied the role of A20 (also known as TNFAIP3), a ubiquitin-editing enzyme that restricts NFκB and cell death signaling, in intestinal homeostasis and tumorigenesis. We have found that A20 expression is consistently reduced in human colonic adenomas than in normal colonic tissues. To further investigate A20’s potential roles in regulating colon carcinogenesis, we have generated mice lacking A20 specifically in intestinal epithelial cells and interbred these with mice harboring a mutation in the adenomatous polyposis coli gene (APC^min^). While A20^FL/FL^ villin-Cre mice exhibit uninflamed intestines without polyps, A20^FL/FL^ villin-Cre APC^min/+^ mice contain far greater numbers and larger colonic polyps than control APC^min^ mice. We find that A20 binds to the β-catenin destruction complex and restricts canonical wnt signaling by supporting ubiquitination and degradation of β-catenin in intestinal epithelial cells. Moreover, acute deletion of A20 from intestinal epithelial cells in vivo leads to enhanced expression of the β-catenin dependent genes cyclinD1 and c-myc, known promoters of colon cancer. Taken together, these findings demonstrate new roles for A20 in restricting β-catenin signaling and preventing colon tumorigenesis.

## Introduction

Colon cancer is the second leading cause of cancer deaths in the USA; hence, understanding its pathogenesis remains a critical goal. Both germ line and somatic mutations in a number of genes have been described to contribute to colon cancer. These mutations contribute to colon carcinogenesis by perturbing cell cycling, cell survival, DNA repair and other critical cellular homeostasis functions [1]. For example, mutations in the adenomatous polyposis coli (APC) gene lead to stabilization of the β-catenin protein, increased transcription of wnt stimulated genes, and perturbed intestinal epithelial cell proliferation and differentiation [2], [3]. In addition, epigenetic factors such as chronic intestinal inflammation increase the risk of malignant transformation [4]–[6]. The link between chronic inflammation and cancer may involve NFκB dependent signals, as continuous activation of the NFκB pathway causes spontaneous intestinal tumors in mice, and intestinal epithelial cell (IEC) specific ablation of IKKβ, protects mice from inflammation associated colon tumors [7], [8].

A20 is a potent anti-inflammatory enzyme, as global loss of A20 leads to multi-organ inflammation and perinatal lethality [9]. A20 performs its anti-inflammatory functions by restricting ubiquitin dependent NFκB and cell death signals [9]–[13]. A20 is a biochemically intriguing protein that exhibits de-ubiquitinating, E3 ligase and ubiquitin binding activities. A20 binds to ubiquitin chains and ubiquitinated signaling complexes and regulates the quantity and type of polyubiquitin chains in these complexes. These chains in turn regulate the activity and stability of signaling proteins such as RIP1, RIP2 and TRAF6[14]–[16]. A20’s anti-inflammatory functions are important for human disease, as genome wide association studies (GWAS) have linked polymorphisms of the A20/TNFAIP3 gene to multiple inflammatory and autoimmune diseases including inflammatory bowel disease[17]–[22]. At least some of these polymorphisms cause reduced expression or function of A20 [23], [24].

While A20’s ability to inhibit NFκB signaling suggests that it may prevent inflammation association malignancies, A20’s capacity to inhibit cell death suggests that it might also promote tumorigenesis by protecting cancer cells from apoptosis. The discovery that bi-allelic somatic mutations of the A20 gene occur in up to 30% of Hodgkin’s lymphoma, MALT, marginal zone, and diffuse large B-cell lymphomas indicated that A20 functions as a tumor suppressor in B cells [25]. However, A20’s NFκB inhibitory functions and cell death inhibitory functions may be integrated differently in distinct cell-types. We have thus investigated A20’s functions in intestinal epithelial cell (IEC) homeostasis and tumorigenesis.

## Materials and Methods

### Antibodies and Reagents

Antibodies directed against A20 (Cell Signaling, 5630; Santa Cruz, sc-376564; Santa Cruz, sc-69980), Active β-catenin (Mllipore, 05–665), Axin (Cell Signaling, 2087), Total β-catenin (Santa Cruz, sc-1496), FLAG (Sigma, F3165), GAPDH (Millipore, MAB374), HA (Santa Cruz, sc-805), MYC (Santa Cruz, sc-789), and pan-ubiquitin (SC-8017) were used for immunoprecipitation and western blot as described below. RKO cells were obtained from the ATCC and maintained in DMEM (Cellgro) supplemented with 10% FCS (Atlanta Biologicals), 1% penicillin-streptomycin-glutamine (Cellgro), and 25 mM HEPES (Cellgro). Recombinant human wnt3a was purchased from R&D systems (5036-WN-010).

### Mice

A20^FL^ mice were generated in our laboratory and described previously [26]. APC^min^ and villin-Cre mice were purchased from JAX. Transgenic mice harboring a tamoxifen-inducible Cre recombinase under the control of the villin-promoter (villin-ER/Cre) were a kind gift from S. Robine (Institut Curie-CNRS, Paris, France). Acute deletion of A20 was performed as previously described [27]. Briefly 1 mg tamoxifen (Sigma, T5648) was dissolved in sterile corn oil and injected intraperitoneally daily for five consecutive days. Tumor number and burden was determined at four months of age with the aid of a stereomicroscope equipped with a sizing reticle (Klarman Rulings, Litchfield NH). All animal studies were conducted in accordance with the University of California, San Francisco Institutional Animal Care and Use Committee.

### Immunoprecipitation and Immunoblotting

Freshly isolated intestinal epithelial cells or RKO cells were lysed in ice-cold 0.5% Triton X-100 (Sigma, BP151) containing 50 mM Tris HCl pH 7.4, 150 mM NaCl, and 10% glycerol and a protease inhibitor cocktail (Roche Complete EDTA-free). For studies concerning ubiquitin modifications, the lysis buffer was also supplemented with 10 mM orthophenanthroline (Sigma, P9375), 10 mM iodoacetamide (Sigma, I6125), and the proteasome inhibitor, MG132 (Enzo, BML-PI102) at a final concentration of 10 uM. The insoluble fraction was removed by centrifugation at 14,000 rpm for 25 minutes in a tabletop centrifuge. Protein concentrations were determined by BCA Assay (Thermo Scientific). For immunoprecipitation, equal amounts of protein were incubated overnight with 3 ug of antibody. Lysates were then immunoprecipitated using protein G Dynabeads (Invitrogen) according to the manufacturers instructions. Samples were resolved on NuPage precast 4–12% Bis-Tris gels (Invitrogen) and transferred to PVDF for western blot analysis.

### Transfection and Luciferase Assays

siRNA to human A20 (5′ GAA GCU CAG AAU CAG AGA UUU 3′) or a control scrambled siRNA (5′ AAC GUA CGC GGA AUA CUU CGA 3′) was purchased from Dharmacon. The MegaTOPFlash luciferase reporter was a kind gift from Dr. Roel Nusse (Stanford University, CA). The pRL-Renilla vector and dual luciferase reporter assay system were obtained from Promega. RKO cells were seeded in 24-well tissue culture plates (Costar) in antibiotic-free media. Twenty-four hours prior to stimulation with recombinant human wnt3a, siRNA (30 pmoles), MegaTOPFlash (500 ng), and Renilla (0.1 ng) was transiently transfected using Lipofectamine (Invitrogen) according to the manufacturers instructions. Twelve hours prior to stimulation with recombinant human wnt3a, cells were cultured in media containing 2% fetal calf serum. Fresh media containing 2% FCS and 150 ng/mL wnt3a was added to the cells for eight hours prior to harvest and determination of luciferase activity. Firefly luciferase activity was normalized to Renilla luciferase activity.

### Plasmids and Cloning

Full-length human A20 and Axin cDNA were obtained from OpenBiosystems. Murine full length A20 was cloned from mouse embryonic fibroblasts. N- and C-terminal truncation mutants were generated by FspI (NEB) digestion. The N-terminal fragment consists of amino acids 1–384 and terminates just prior to zinc finger 1. The C-terminal mutant consists of amino acids 369–775 and begins just prior to zinc finger 1. Full-length human and mouse, truncated murine A20 constructs and Axin were cloned into pCMV-3Tag (Agilent) vectors containing a N-terminal 3FLAG or 3MYC epitope tag respectively. A20 deficient RKO cells were generated by TALEN technology [28]. TALEN targeting sequences are available upon request.

### Primary Intestinal Epithelial Cell Isolation and Quantitative PCR

Murine intestinal epithelial cells were isolated using the protocol of G.S. Evans with some modifications [29]. Briefly, intestines were excised and placed in ice-cold HBSS (Cellgro) containing 2% glucose (Sigma). Intestines were flushed with HBSS containing 1 mM DTT to remove fecal contents and then opened along their antimesenteric side. Intestinal segments were cut into 2–3 mm sections and then washed five times in ice-cold HBSS with 2% glucose. After washing, intestinal sections were incubated with Ca^2+^ and Mg^2+^ free PBS containing 10 mM EDTA for 30 minutes at room temperature with gentle agitation. After incubation, samples were agitated briefly to release epithelial cells. The supernatant, containing enriched epithelial cells, was collected and washed in ice-cold HBSS with 2% glucose.

Total RNA was isolated by Trizol (Invitrogen) according to the manufacturers instructions. One microgram of total RNA was used for reverse transcription using the Quantitech Reverse Transcription kit (Qiagen). Quantitative PCR was performed using the following Taqman primers (Applied Biosystems, Carlsbad CA): TNFAIP3 (Mm00437122_m1), Cyclin D1 (Mm00432359_m1), MYC (Mm00487803_m1), HPRT1 (Mm00446968_m1). Quantitative PCR was performed using the Gene Expression Master Mix (Applied Biosystems, 4369016) on an ABI 7300 using the following protocol: 50° 2 min, 95° 10 min, 95° 15 sec, 60° 1 min for 40 cycles. Ct values were normalized to HPRT1 values.

### Immunohistochemistry

Resected intestinal tissue was fixed in 10% neutral-buffered formalin (Sigma) overnight. Tissue specimens were then embedded in paraffin and stained according to standard protocols.

### Statistical Analysis

Statistical analysis was performed with Graphpad Prism 4 (Graphpad Software, San Diego, CA). Comparisons between two groups were performed by two-tailed unpaired Student’s t-test. Multigroup comparisons were performed by one-way analysis of variance. P<0.05 was used as the threshold for statistical significance.

## Results and Discussion

**Figure 1:**
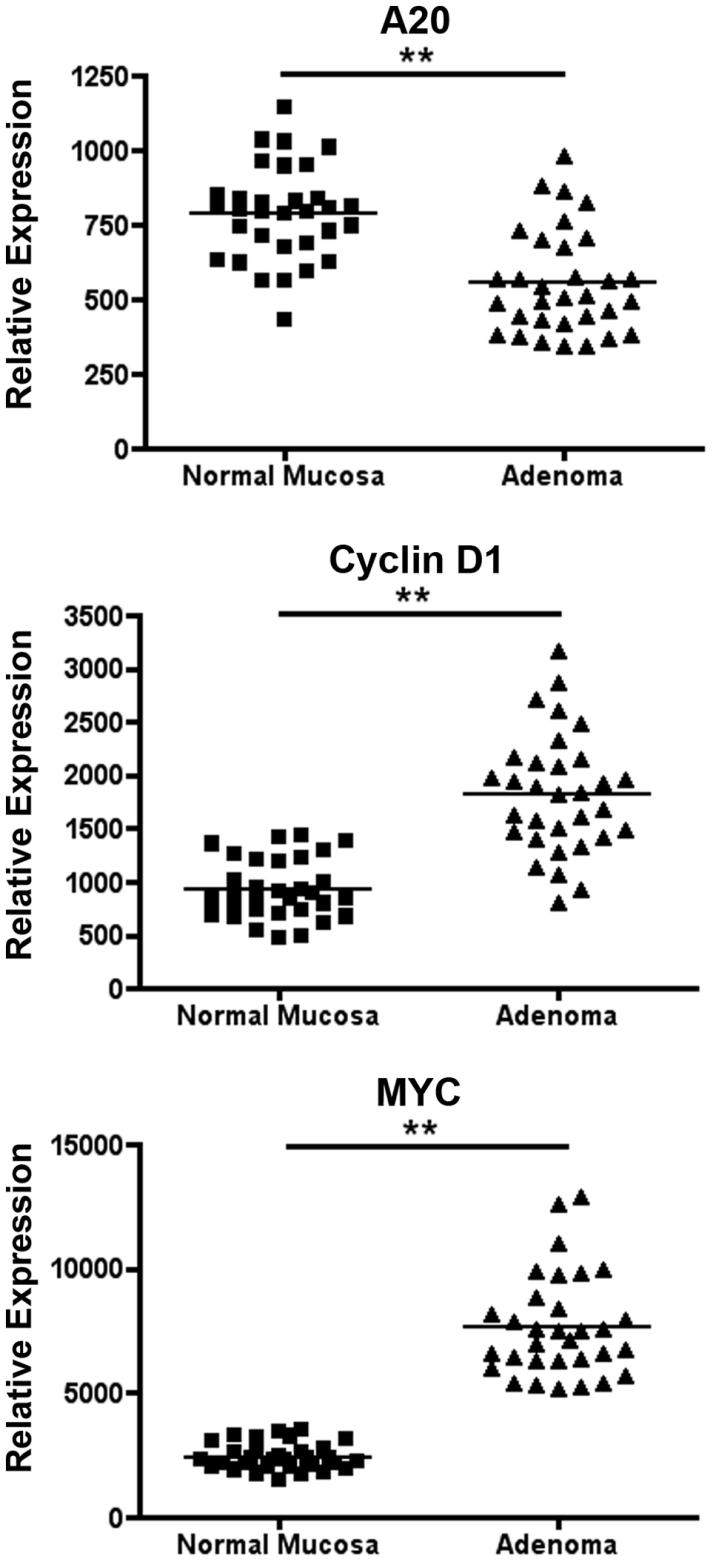
Human colonic adenomas express less A20 than normal colonic mucosa. Expression of A20 (top panel), cyclin D1 (middle panel) and c-myc (bottom panel) mRNAs in normal colonic mucosa and colonic adenomas, as quantitated by the Genome Expression Omnibus (GDS2947). Relative expression levels are shown. **indicates p<0.01.

**Figure 2:**
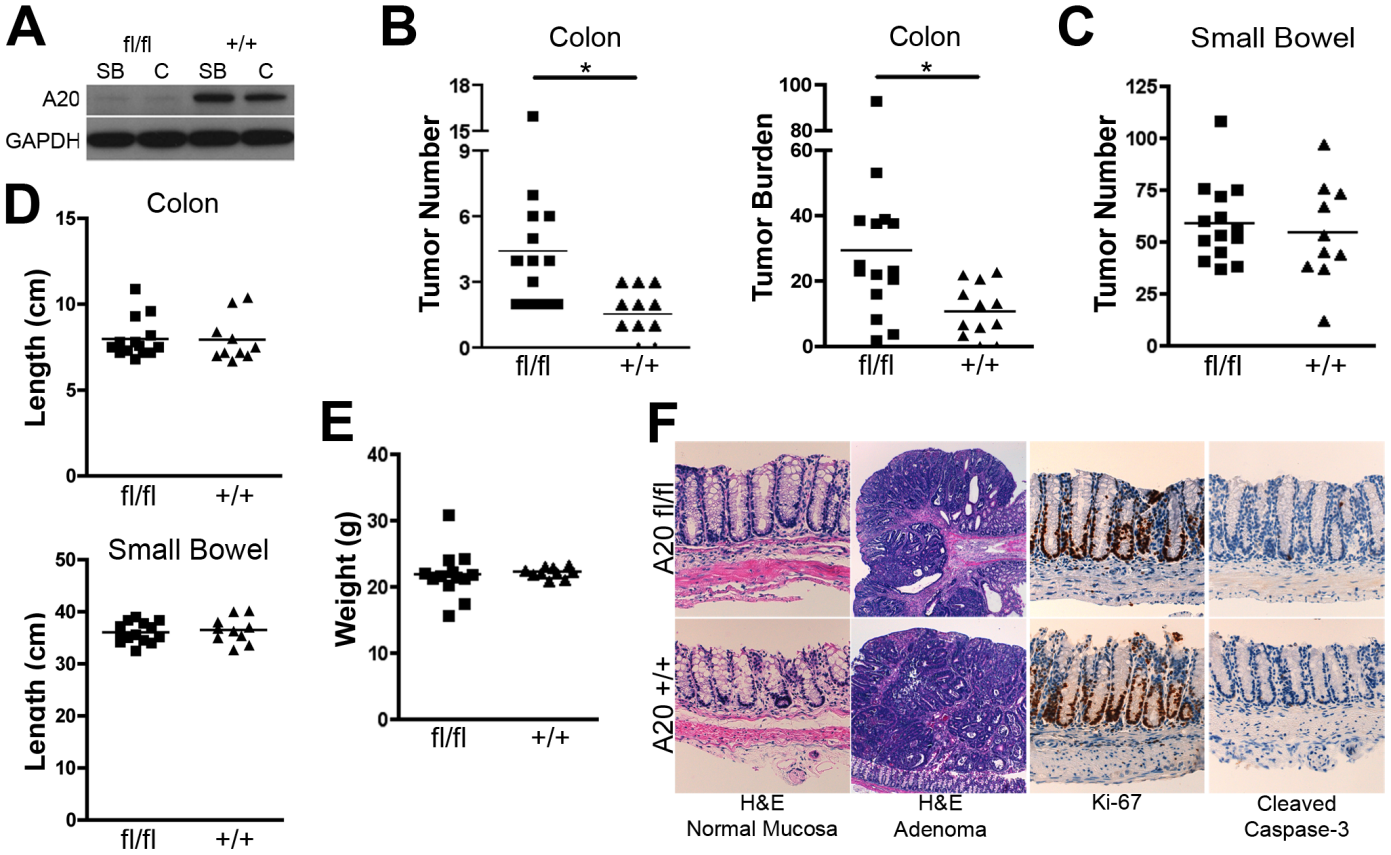
A20 expression in IECs restricts colon tumorigenesis. (A) immunoblot analysis of isolated IECs indicating efficient deletion of A20 from the small bowel (SB) and colon (C) of villin-Cre A20^FL/FL^ APC^min/+^ mice (fl/fl) compared to control villin-Cre A20^+/+^ APC^min/+^ mice (+/+) mice. GAPDH is shown as a loading control. (B) Tumor number (left panel) and aggregate tumor size (right panel) in colons of A20 (fl/fl) and wild-type (+/+) mice harboring APC^min^ mutation. (C) Tumor numbers in small intestines of A20 (fl/fl) and wild-type (+/+) mice harboring APC^min^ mutation. (D) Colon and small intestine lengths from (fl/fl) and wild-type (+/+) mice harboring APC^min^ mutation. Each point represents one mouse. Lines indicate mean values. (f) Hematoxylin and eosin staining (upper panels) and Ki-67 and cleaved caspase-3 immunostaining (lower panels) of colonic sections from villin-Cre A20^FL/FL^ APC^min/+^ mice (fl/fl) and control villin-Cre A20^+/+^ APC^min/+^ mice. 40X magnification shown.

We first addressed potential epigenetic changes in A20 expression in colon carcinogenesis by searching NCBI’s Genome Expression Omnibus [30], [31]. We found that A20 mRNA expression is significantly decreased in human adenomatous colonic polyps as compared to normal surrounding tissue (Fig. 1, Fig. S1). These human colon tumors also express increased levels of the cell proliferation related genes c-myc and cyclinD1 (Fig. 1, Figure S1). Several recent reports have also suggested genetic and epistatic changes in A20 expression in human colorectal cancer [32], [33]. To directly test A20’s potential cell-autonomous functions in IECs, we generated mice lacking A20 specifically in these cells by interbreeding A20^FL^ mice with villin-Cre transgenic mice [26]. Compound A20^FL/FL^ villin-Cre mice effectively eliminated A20 protein expression from both small intestinal and colonic epithelial cells (Fig. 2A). Previous reports have shown that mice harboring an intestinal epithelial cell-specific deletion of A20 were grossly normal and did not exhibit spontaneous inflammation or tumor development [34]. However, these mice were more susceptible to induced models of colitis. Conversely, overexpression of A20 in intestinal epithelial cells protects mice from dextran sodium sulfate induced colitis, potentially through an enhancement of the epithelial barrier [35], [36]. To determine whether A20 expression might predispose to colon carcinogenesis, we interbred A20^FL/FL^ villin-Cre mice with APC^min^ mice that harbor a mutation in the APC gene [37]. Heterozygous APC^min/+^ mice develop spontaneous intestinal tumors, while homozygous APC^min/min^ mice are embryonically lethal [37], [38]. Importantly, this model does not involve gross inflammation such as that induced by repeated dextran sulfate sodium (DSS) and the mutagen azoxymethane (AOM). As previously reported, A20^+/+^ APC^min/+^ mice spontaneously developed multiple small intestinal tumors but infrequent colonic tumors (Fig. 2B, 2C). Remarkably, A20^FL/FL^ villin-Cre APC^min/+^ mice possessed significantly increased numbers of colonic adenomas when compared to A20^+/+^ villin-Cre APC^min/+^ mice (Fig. 2B). The aggregate size of colonic polyps was also increased in these mice (Fig. 2B). The number of small bowel polyps was not affected by A20 deficiency in small intestinal IECs (Fig. 2C). Thus, A20 appears to regulate colonic tumorigenesis.

As A20 restricts NFκB signaling, A20 might restrict colon tumorigenesis by preventing spontaneous intestinal inflammation in A20^FL/FL^ villin-Cre APC^min/+^ mice. However, similar to A20^FL/FL^ villin-Cre and A20^+/+^ villin-Cre mice, no signs of spontaneous inflammation were present in A20^FL/FL^ villin-Cre APC^min/+^ and A20^+/+^ villin-Cre APC^min/+^ mice, and there were no differences in intestinal length or mouse weights (Fig. 2D, 2E). Histologic examination of colonic neoplasms and surrounding normal intestinal tissues did not reveal significant differences between A20^FL/FL^ villin-Cre APC^min/+^ and A20^+/+^ villin-Cre APC^min/+^ mice in inflammation, apoptosis, or proliferation rates (Fig. 2F). Thus, A20 expression in IECs does not strongly restrict basal intestinal inflammation.

The APC gene is mutated in the majority of sporadic colorectal adenocarcinomas [39] and in nearly all cases of familial adenomatous polyposis [40], [41]. The APC protein is part of a multi-protein complex termed the destruction complex. This complex, including the E3 ligase complex SCF/β-TRCP, causes phosphorylation, ubiquitination, and degradation of β-catenin [42]. Stimulation of cells with wnt ligands, or mutations of proteins such as APC, interrupt this process, allowing β-catenin to migrate to the nucleus and induce transcription of β-catenin dependent genes [3], [43]. Recently, renewed interest has arisen concerning ubiquitination of the components of the classical wnt/β-catenin signaling cascade as a mechanism for its regulation [44].

**Figure 3:**
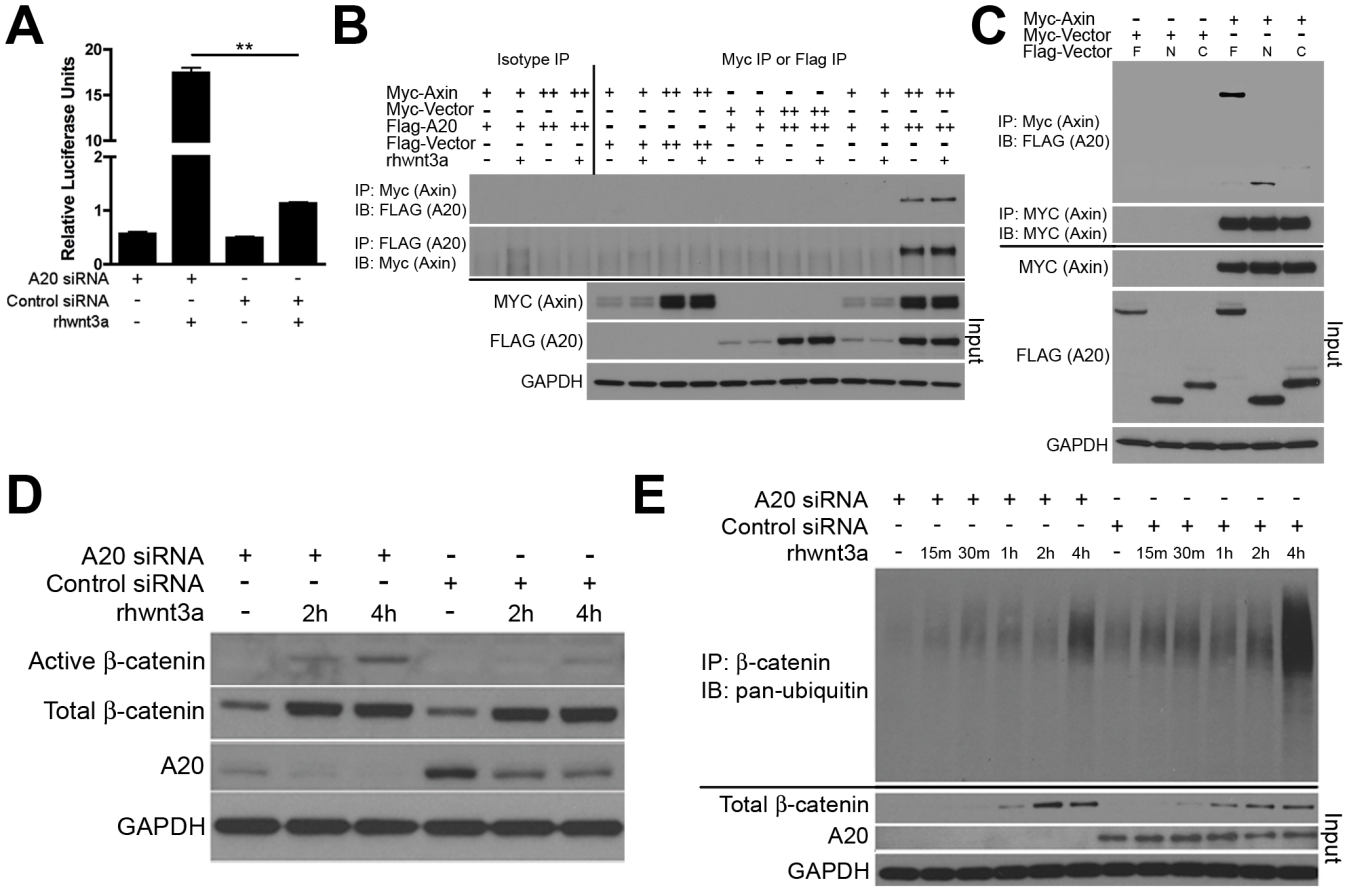
A20 supports β-catenin ubiquitination and degradation through an interaction with the destruction box. (A) Luciferase assay showing transcriptional activity of a β-catenin dependent TCF/LEF4 reporter in RKO cells. Cells were treated with A20 specific or control siRNA and recombinant human wnt3a (rhwnt3a) as indicated. Relative luciferase units (RLU) are shown. **indicates p<0.01. (B) Co-precipitation of A20 with Axin. RKO cells transfected with the indicated expression plasmids were lysed, immunoprecipiated (IP) for the indicated epitope tag, and immunoblotted (IB) for the indicated proteins. Cells were stimulated with rhwnt3a or control as indicated for four hours. Input levels of MYC, FLAG, and GAPDH are shown as controls below. (C) Co-precipitation of partial A20 proteins with Axin. Co-transfection experiments as in (B). Input levels of MYC, FLAG, and GAPDH shown below. (D) A20 suppresses wnt3a stimulated induction of β-catenin expression. Immunoblot analyses of active and total β-catenin expression in RKO cells treated with A20 specific or control siRNA. A20 and GAPDH levels shown below as loading control. (E) A20 supports wnt3a stimulated β-catenin ubiquitination. RKO cells were treated with A20 specific or control siRNAs and wnt3a for the indicated times. Lysates were immunoprecipitated for β-catenin followed by immunoblotting for ubiquitin. Input amounts of beta-catenin, A20, and GAPDH proteins shown below as controls. All data are representative of three or more independent experiments.

Given A20’s pleiotropic functions in regulating ubiquitin dependent signals, we hypothesized that A20 might regulate wnt signaling. To investigate this possibility, we first used an siRNA approach to reduce A20 expression in RKO cells, a cell line with intact wnt induced β-catenin signaling [45]. Acute knockdown of A20 expression caused a marked increase in TCF/LEF4 luciferase reporter activity after recombinant human wnt3a stimulation when compared to cells transfected with control siRNA (Fig. 3A). These results suggest that A20 directly restricts wnt induced signaling.

To better understand how A20 restricts β-catenin signaling, we investigated whether A20 binds to destruction complex proteins that regulate β-catenin ubiquitination. A20 co-precipitated with both heterologously expressed and endogenous Axin, the scaffolding protein that binds many destruction complex proteins (Fig. 3B, Figure S2). No change in this association was seen after wnt3a stimulation, suggesting a constitutive association between these two molecules. A20 utilizes a number of motifs to bind ubiquitin chains, other ubiquitin binding proteins, and other ubiquitin editing enzymes [46]. We thus tested whether A20’s N-terminal or C-terminal domains mediate binding of A20 to Axin. Full length A20 co-precipitated with Axin, as did (to a lesser extent) the N-terminal OTU domain of A20 (Fig. 3C). By contrast, the C-terminal portion of A20 containing its seven zinc fingers failed to co-precipitate with Axin, suggesting that A20 preferentially utilizes its N-terminal OTU domain to bind Axin (Fig. 3C). These studies suggest that A20 interacts directly with the β-catenin destruction complex.

As β-catenin protein levels are tightly regulated by the SCF/β-TRCP E3 ligase complex, we tested the role of A20 in regulating β-catenin ubiquitination and stability. Stimulation of both A20 siRNA and control siRNA knockdown RKO cells with wnt3a led to increased levels of total β-catenin, however, A20 deficient RKO cells accumulated significantly greater levels of the active dephosphorylated form of β-catenin when compared to control RKO cells (Fig. 3D). Notably, A20 protein levels did not increase after wnt3a stimulation, suggesting that wnt3a does not stimulate NFκB dependent induction of A20 expression (Fig. 3D). In addition, increased β-catenin dependent TCF/LEF4 reporter activity in A20 deficient RKO cells did not result from increased β-catenin mRNA expression, suggesting that A20 regulates β-catenin via post-transcriptional or post-translational mechanisms (Fig. S3). Cellular β-catenin protein levels are regulated at the post-translation level through K48-linked ubiquitination and subsequent proteasomal degradation. To determine whether A20 regulates β-catenin ubiquitination, we stimulated RKO cells with wnt3a ligand, immunoprecipitated β-catenin, and immunoblotted for ubiquitin. A20 deficient cells accumulated significantly decreased levels of ubiquitinated β-catenin than control cells, particularly at four hours after wnt3a stimulation (Fig. 3E). These results correspond with increased β-catenin levels in A20 deficient cells (Fig. 3E). Taken together, these studies indicate that A20 binds to the APC destruction complex and restricts wnt3a induced β-catenin signaling by promoting β-catenin ubiquitination and degradation.

**Figure 4:**
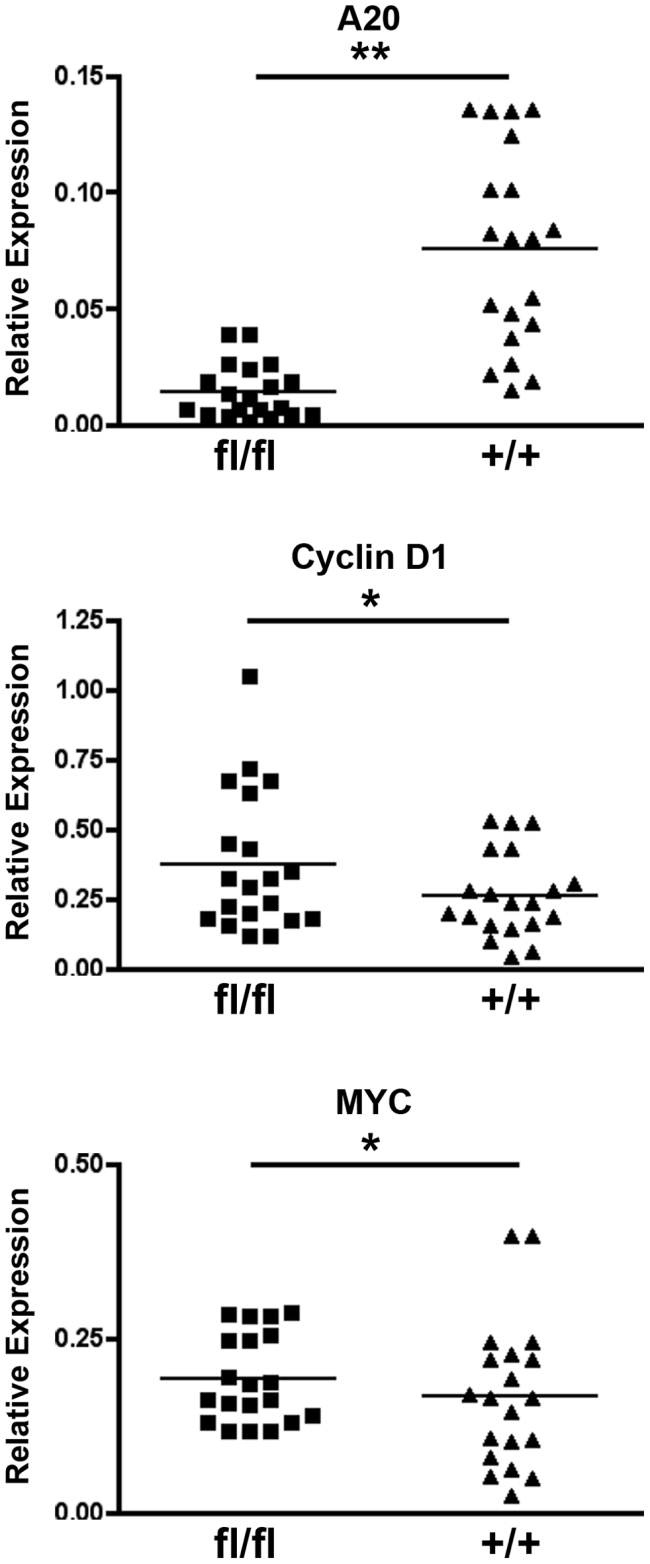
Acute deletion of A20 from IECs leads to increased levels of Cyclin D1 and MYC mRNA in vivo. Villin-ER/Cre A20^FL/FL^ (fl/fl) and control Villin-ER/Cre A20^+/+^ (+/+) were injected with 1 mg of tamoxifen daily for 5 days. IECs were then isolated and studied for expression of A20 (upper panel), Cyclin D1 (middle panel), and MYC (lower panel) mRNAs by qPCR. Each point represents one mouse. *indicates p<0.05; **indicates p<0.01.

Finally, to confirm that A20 expression in IECs regulates wnt/β-catenin signaling in vivo, we interbred A20^FL/FL^ mice with mice expressing a tamoxifen-sensitive Cre recombinase under the villin gene promoter elements (villin-ER/Cre) [47]. Intra-peritoneal injection of tamoxifen into compound A20^FL/FL^ villin-ER/Cre and control A20^+/+^ villin-ER/Cre mice led to reduction of A20 mRNA expression and increased expression of the β-catenin dependent cyclinD1 and c-myc mRNAs in purified intestinal epithelial cells (Fig. 4). These genes are also increased in human colonic adenomas (Fig. S1). Thus, A20 restricts β-catenin dependent signaling in vitro and in vivo.

In summary, we have discovered that the ubiquitin-modifying enzyme A20 is a tumor suppressor for colon carcinogenesis. This tumor suppressor function appears to be due to a novel function for A20 in restricting wnt induced β-catenin signaling. The relevance of our findings to human disease is high, as reduced A20 expression is a common epigenetic finding in human adenomas, and as the increased tumorigenesis of A20^FL/FL^ APC^min/+^ mice appears to be selective for colonic tissue, where most human intestinal cancers occur. Our findings extend A20’s tumor suppressor function from B cell lymphomas to colon cancers. Finally, these studies demonstrate that A20, a potent regulator of multiple human autoimmune and inflammatory diseases, is also broadly important for preventing malignant transformation.

## Supporting Information

Figure S1 **Decreased A20 expression correlates with increased Cyclin D1 and MYC expression in colonic adenomas obtained from patients compared to surrounding normal mucosa.** Data derived from the Genome Expression Omnibus (GDS2947).
(TIF)

Figure S2 **Endogenous A20 Interaction with Axin.** A) A20 deficient RKO cells generated by TALEN technology (left) or wild-type RKO cells (right) were stimulated with TNF-α for two hours prior to lysis. Immunoprecipitation of endogenous A20 using either the B5 or 4H16 antibody from Santa Cruz Biotechnology efficiently co-immunoprecipitated endogenous Axin. Inputs are shown below. GAPDH is shown as a loading control. B) MYC tagged Axin or MYC-tagged vector control was co-expressed with FLAG-tagged A20 or FLAG-tagged vector control. Immunoprecipitation was performed with anti-Axin antibody and western blot was performed with the anti-FLAG antibody. Input levels of FLAG, MYC, and GAPDH proteins shown below as controls.
(TIF)

Figure S3 **A20 does not suppress β-catenin mRNA expression.** RKO cells transfected with either A20 specific or control siRNAs were stimulated with wnt3a for the indicated times. Total RNA was isolated and subjected to qPCR analysis for β-catenin expression. Positive control is a plasmid expressing β-catenin.
(TIF)
