# Supplementary figures and images for: A20 Restricts Wnt Signaling in Intestinal Epithelial Cells and Suppresses Colon Carcinogenesis
Source: PLoS One. 2013 May 6;8(5):e62223. doi: 10.1371/journal.pone.0062223 (PMC3645994; doi:10.1371/journal.pone.0062223)

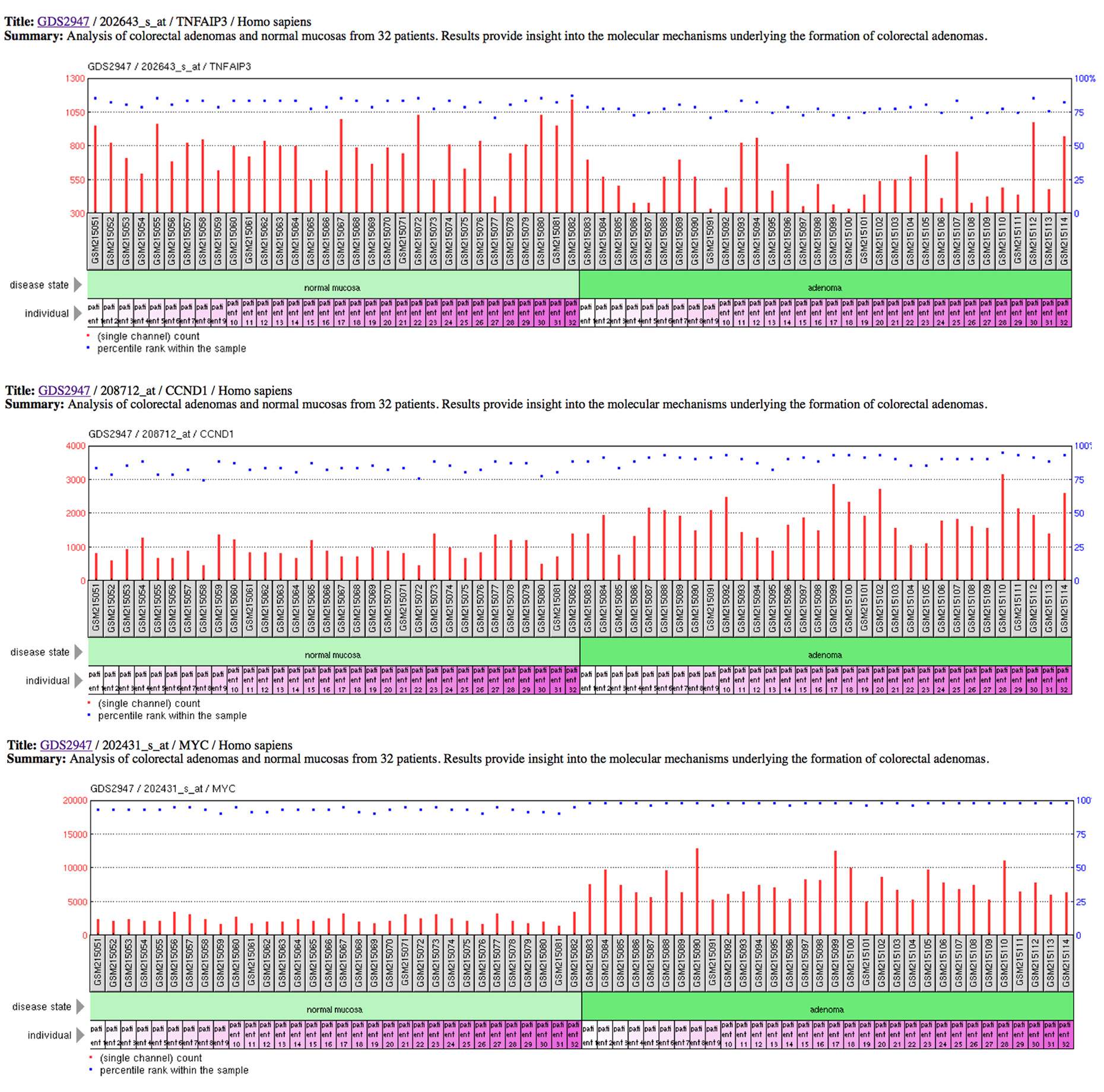

Supplement: Figure S1 — Decreased A20 expression correlates with increased Cyclin D1 and MYC expression in colonic adenomas obtained from patients compared to surrounding normal mucosa. Data derived from the Genome Expression Omnibus (GDS2947). (TIF) [file pone.0062223.s001.tif]

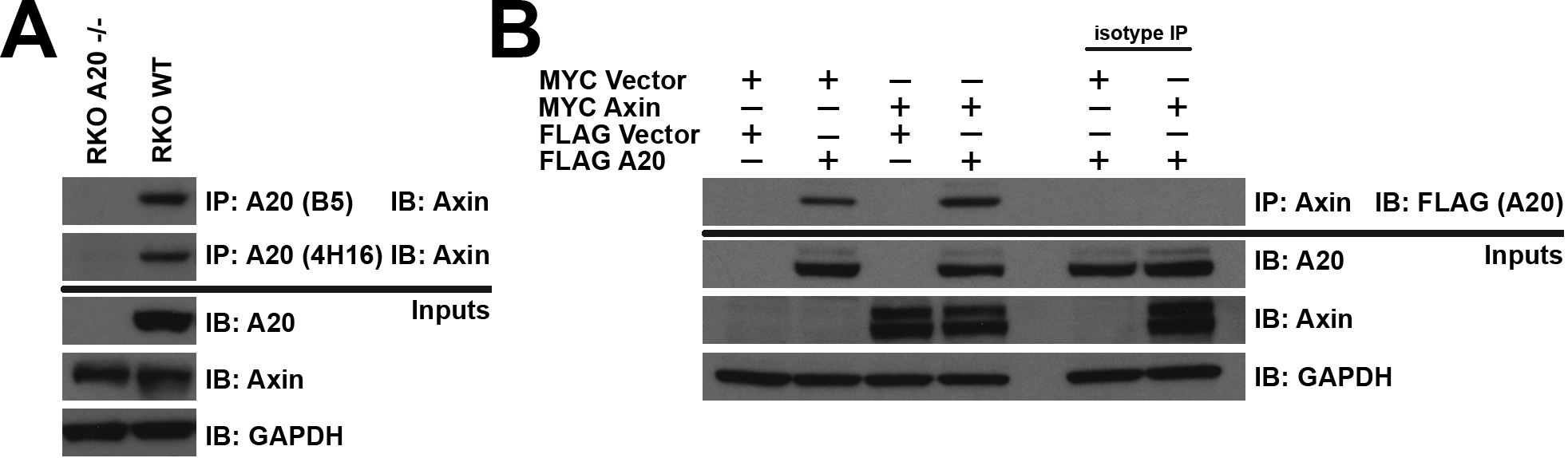

Supplement: Figure S2 — Endogenous A20 Interaction with Axin. A) A20 deficient RKO cells generated by TALEN technology (left) or wild-type RKO cells (right) were stimulated with TNF-α for two hours prior to lysis. Immunoprecipitation of endogenous A20 using either the B5 or 4H16 antibody from Santa Cruz Biotechnology efficiently co-immunoprecipitated endogenous Axin. Inputs are shown below. GAPDH is shown as a loading control. B) MYC tagged Axin or MYC-tagged vector control was co-expressed with FLAG-tagged A20 or FLAG-tagged vector control. Immunoprecipitation was performed with anti-Axin antibody and western blot was performed with the anti-FLAG antibody. Input levels of FLAG, MYC, and GAPDH proteins shown below as controls. (TIF) [file pone.0062223.s002.tif]

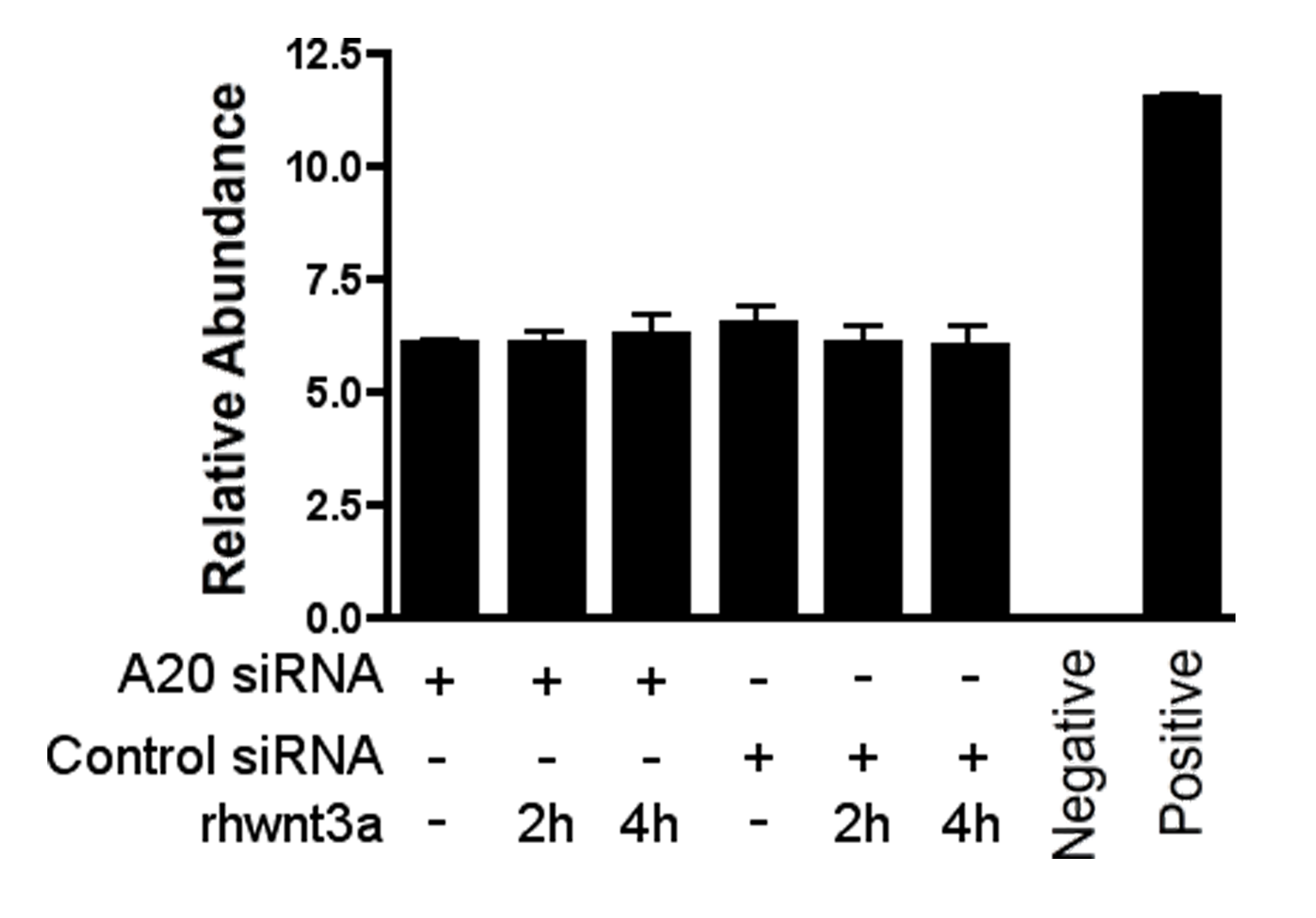

Supplement: Figure S3 — A20 does not suppress β-catenin mRNA expression. RKO cells transfected with either A20 specific or control siRNAs were stimulated with wnt3a for the indicated times. Total RNA was isolated and subjected to qPCR analysis for β-catenin expression. Positive control is a plasmid expressing β-catenin. (TIF) [file pone.0062223.s003.tif]
